# Timing and Cost Consideration in Surgery for Male Stress Urinary Incontinence For Current Urology Reports

**DOI:** 10.1007/s11934-026-01367-8

**Published:** 2026-09-23

**Authors:** Eric Chung, Charles Yizhou Ruan, Juan Wang

**Affiliations:** 1 AndroUrology Centre, QLD and Sydney, Brisbane, NSW Australia; 2 University of Queensland, Princess Alexandra Hospital, Brisbane, QLD Australia; 3 Macquarie University Hospital, Sydney, NSW Australia; 4 AndroUrology Centre, Suite 3, 530 Boundary St , Brisbane, QLD 4000 Australia

**Keywords:** Urinary incontinence, Surgery, Male sling, Artificial urinary sphincter

## Abstract

**Purpose of Review:**

Surgery remains the standard of definitive care in male stress urinary incontinence (SUI). This narrative review provides a critical discussion of pertinent aspects of male continence surgery, including cost-benefit analysis and whether continence surgery should be offered earlier.

**Recent Findings:**

Controversies exist regarding the timing of surgery and whether the male sling (MS) is superior to the artificial urinary sphincter (AUS). Presently, significant gaps exist in the cost analysis and modelling for the most effective treatment in male SUI. Each continence device has its own merit and drawbacks, and patients should be counselled on the procedure, mechanics, expectations and surgical complications, with emphasis on shared decision-making and personalised care.

**Summary:**

More high-quality studies of surgical interventions, including direct comparison against non-surgical methods, coupled with in-depth evaluation on why patients choose a particular treatment over another, will be useful in preoperative counselling and streamlining decision-making in male SUI.

## Introduction

Male stress urinary incontinence (SUI) is more common in males who have undergone treatment for prostate cancer [1–3]. It is estimated that more than 10% of patients will suffer from post-prostatectomy SUI at 12 months despite advances in surgical techniques and robotic technology [2, 4, 5]. While direct damage to the external urethral sphincter is the most common cause for post-prostatectomy SUI, various factors can influence the degree of continence, such as nerve-sparing surgery, radiation therapy, older age, pre-existing intrinsic sphincter deficiency, concomitant de novo bladder dysfunction and presence of underlying cardiometabolic disorders [6]. On the other hand, radiation-induced SUI tends to occur later but is often more severe and refractory to conservative measures, highlighting that the mechanism of radiation injury causes a likely “fibrosed” proximal urethra and bladder neck coupled with a higher likelihood of (secondary) bladder dysfunction [7, 8].

The presence of SUI affects various quality of life (QOL) domains in the home, workplace and social outings [9–11]. Literature shows that SUI can be associated with emotional distress, depression and anxiety, as well as resulting in the lack of interest in or fear of leaving the house, going to work or engaging in social activities [12, 13]. The economic burden of SUI is estimated to be in the billions annually, including direct and indirect productivity losses [14, 15]. While the exact magnitude of these indirect costs is unknown, they can lead to changes in work schedule, leave from work or be related to disability, and potentially retirement at an earlier stage. Many patients are reluctant to seek treatment due to embarrassment, stigma, cost or fear of treatment [10, 11]. SUI is often considered a social taboo in many societies; SUI results in self-imposed social isolation [10]. This invariably leads to significant psychological distress with ensuing feelings of shame, depression, loss of self-esteem, and apathy. Furthermore, literature shows that SUI negatively impact sexuality, and many patients experience poor confidence, unattractiveness, and stigmatisation, further compounding the existing sense of loneliness, self-isolation and depression [13, 16, 17].

The treatment algorithm for male SUI centres on early pelvic floor exercises (PFE) to restore the continence mechanism, and if patients fail conservative measures, surgical intervention provides the most definitive and prompt treatment [1, 2]. Conservative measures such as incontinence pads, condom catheters, and various penile clamps do not result in a permanent solution for SUI. Surgery for male SUI is largely divided into male sling (MS) or artificial urinary sphincter (AUS), or similar sphincteric devices [18]. In recent times, synthetic MS is becoming more popular, given that it is cheaper than AUS, relatively straightforward surgery and that patients do not need to operate a device to void. The MS can be classified based on non-adjustable or adjustable, each with its own specific nuances and unique adverse events. The ideal candidate for MS should have a mild to moderate degree of SUI with adequate residual sphincter function and sufficient bladder pressure to void against the “obstructive” MS. In contrast, the AUS requires physical manipulation of the “sphincter device” to void and is traditionally reserved for patients with severe incontinence and those who have failed MS surgery. The following article provides a narrative critical discussion regarding pertinent factors involved in surgical timing and cost-benefit considerations in male SUI surgery.

## Methods and Materials

A literature review using the PubMed database was performed to identify peer-reviewed, English-language articles published pertaining to the management of male SUI with an emphasis on the timing of surgical intervention and the cost-effectiveness analysis of various male continence devices. Given the lack of proper randomised controlled trials and direct comparative trials between the various continence devices, a formal PRISMA format is not adopted, and this article is provided as a narrative review. This review is not designed to address the different types of male continence procedures nor discuss the various complications that can arise from each continence procedure.

## Discussion

### Rationale For Non-Surgical Options in Male SUI

Conservative measures in SUI encompass behavioural modifications with fluid restriction and timed voiding, PFE, and the use of incontinence pads, condoms, catheters, and/or penile clamps [2]. While a urinary catheter can be “effective” for SUI, it is associated with various issues (catheter bypass, need for 4 to weekly change, cost, etc.) and complications (urinary tract infections, bleeding, pain, etc.). On the other hand, a penile clamp is an easy and likely cost-effective strategy to maintain continence and avoid the need for an indwelling catheter and its drawbacks. Various penile clamps can be readily purchased online, although some devices have poor design or materials [19]. However, it may not be suitable for every patient and can cause discomfort to the penis from excessive pressure with constant penile clamp use. Penile clamps have a higher occlusive pressure and are more likely to produce a higher continence level, but they can cause greater local (penile) pain. Biomechanics studies have shown that penile clamps can decrease the blood flow and tissue perfusion in the penis and urethra, although the increases in inflammation often resolve after removal of the device [20]. It is generally recommended that patients not wear the penile clamp continuously to avoid pressure necrosis (or strangulation) of the penis. Real-world data on the use of these mechanical non-surgical devices [21] showed that while advances have been made in urinary clamps over the years, there is still a need to innovate for better clamp comfort for wearers.

Literature supports the role of pre-treatment PFE to improve the continence level by continuously strengthening the underlying pelvic floor muscles and fascia, to maintain and increase the distal urethral sphincter tension and volume, thus, the ability to control urination [22–27]. Targeted PFE can improve pelvic floor muscle contractility and enhance urethral sphincter function [28–31]. Nonetheless, preoperative PFME with or without biofeedback remains controversial due to conflicting results and the heterogeneity in PFE regimes with inconsistent feedback methods, including visual, oral, probe and surface electrodes [25, 32–34]. Nonetheless, a systematic review with meta-analysis exploring various factors that differentiate successful from unsuccessful patient outcomes in post-prostatectomy SUI found that preoperative PFE, concomitant use of biofeedback, and specific urethral instructions are associated with successful patient outcomes [35].

### Should Continence Surgery Be Offered and Performed Earlier During The Postoperative (Recovery) Period?

While PFE can be effective in preserving and restoring continence following radical prostatectomy, it is estimated that around 10% of patients will live with SUI at 12 months postoperative [36]. The data on SUI post-radiation therapy is generally worse, especially if the post-prostatectomy patients received adjuvant or salvage radiation therapy [37, 38]. Furthermore, the exact role of PFE in the radiation cohort remains uncertain, given secondary bladder dysfunction complicating the SUI presentation [39]. Additionally, the use of concurrent androgen deprivation therapy further weakened the pelvic floor muscles [40].

Another meta-analysis on preoperative PFE reported that PFE significantly lowered the incidence of early postoperative SUI only, with no significant difference observed during the perioperative PFE and postoperative PFE groups in recovery of SUI, with OR of 0.58 (95%CI: 0.20–1.71), 0.58 (95%CI:0.20–0.71) and 0.66 (95%CI: 0.32–1.38) in 1, 3 and 6 months after surgery [41]. A more recent systematic review and meta-analysis including more than 1365 patients over twelve studies [42], concluded that preoperative PFE can improve postoperative urinary incontinence at 3 months post- prostatectomy (OR: 0.61; 95% CI, 0.37–0.98, *p* = 0.04), but it cannot improve urinary incontinence at 6 months (OR: 0.57; 95% CI, 0.28–1.17, *p* = 0.13) or longer after surgery, suggesting that preoperative PFE can improve early continence rate, but cannot improve long-term urinary continence rate.

The Cochrane review involving 3079 participants across 25 studies [43] also confirmed that combined PFE and biofeedback result in minimal change in the subjectively cured or improved of incontinence rates between 6 and 12 months (RR 0.97, 95% CI 0.79 to 1.19; 2 studies; *n* = 788; low-certainty evidence; in absolute terms: no treatment or sham arm: 307 per 1000 and intervention arm: 297 per 1000). Interestingly, there is little difference reported in terms of objective cure or improvement of incontinence between 6 and 12 months (MD 0.18, 95% CI -0.24 to 0.60; 2 studies; *n* = 565; high-certainty evidence) between combinations of conservative treatments and control, although men in the combined treatments group experienced greater muscle-related adverse events (RR 2.92, 95% CI 0.31 to 27.41; 2 studies; *n* = 136; very low-certainty evidence; in absolute terms: 0 per 1000 for both arms).

There is a lack of standardisation in PFE techniques, with considerable variations in protocols concerning combinations of conservative treatments to advocate effective PFE as a single effective treatment. Nonetheless, those with severe SUI or those with SUI following radiation therapy are unlikely to improve with PFE [2, 39]. Given the lack of significant improvements in SUI after 3 months, coupled with the significant psychosocial and sexual distress experienced in patients with SUI, an argument can be made that patients who are not continent by 3 months or bothered by a significant degree of SUI and wish to undergo definitive therapy should be advised to undergo surgical intervention to restore continence.

Radiation therapy invariably worsens SUI and can present surgical challenges due to high urethral stricture rates, a fibrosed urethral sphincter and potentially greater risk of device-related erosion. Discussion on the timing of continence surgery in relation to radiation therapy is often dictated by the severity of incontinence, urgency to commence radiation therapy and desire for earlier (or later) intervention. The advantages of proceeding with an earlier continence surgery before radiation therapy are earlier restoration and preservation of continence (against worsening SUI following radiation), as well as the option of going for a male sling. On the other hand, delaying surgery until after radiation therapy allows the patient (and surgeon) to see how bad the incontinence will become and potentially avoid having to undergo a second continence surgery (in the setting of recurrent SUI after radiation therapy). The AUS is considered the standard of care for radiation-related SUI, and a transcorporal cuff placement can minimize the risk of cuff erosion in the irradiated urethra [1, 2].

### Contemporary Surgical Devices For Male SUI Following Failed Conservative Measures

The use of a periurethral bulking agent in male SUI is not generally considered a standard of care, with its role debatable regarding the optimal agent, patient selection, and technique(s) [2]. Published literature shows suboptimal clinical outcomes compared to other surgical continence treatments, with low cure rates and the need for multiple and/or repeated periurethral injections, as well as the risks of subsequent deterioration and/or migration of bulking particles over time [44, 45].

Commercially successful adjustable MS are Argus (Promedon, Cordoba, Argentina), ReMeex (Neomedic, Barcelona, Spain) and ATOMS (AMI, Feldkirch, Austria), while current non-adjustable MS are Advance (Boston Scientific, Minnetonka, USA) and Virtue (Coloplast, Minneapolis, USA) slings [46]. While the adjustable MS can be revised in the room or outpatient by adding additional fluid in the system without the need for a proper surgery, there is no strong evidence to suggest that adjustable MS is superior to non-adjustable MS [2, 46]. In a study comparing adjustable vs. non-adjustable MS, the continence and patient satisfaction rates were quite similar despite more men choosing the adjustable MS [47].

The AMS 800 (Boston Scientific, Minnetonka, USA) has shown long-term records of safety, durability and clinical efficacy [48, 49]. More recently, ContiClassic and ContiReflex AUS devices (Rigicon Inc., NY, USA) have been introduced to provide a greater selection of pre-set cuff diameter sizes and the ability to adapt the occlusive (urethral) cuff pressure in a real-time manner against any sudden increase in intra-abdominal pressure changes [50]. When given the choice between MS and AUS, most men with post-prostatectomy SUI preferred MS for earlier return to continence since they do not need to manipulate a device to void [51]. Nonetheless, it is important to understand the different mechanisms of action between MS and AUS. The MS creates a “hammock-like” obstruction to increase the urethral coaptation and resistance, while AUS provides a circumferential occlusion of the urethra [18]. The AMS 800 device is often used to salvage male sling failure cases, with the placement of AUS not an issue most of the time [50]. It is rare for a patient to receive a male sling after an AUS failure.

The recent consensus findings from the 7th International Consultation on Incontinence have concluded that an AUS is the preferred treatment for properly selected men who have moderate to severe stress incontinence after radical prostatectomy, as the AUS has the longest record of safety and efficacy, although exposure to radiation therapy is considered an adverse risk factor with higher complication rates [2]. MS provides a valid and acceptable surgical alternative with intermediate follow-up data supporting its safety and efficacy in men with mild to moderate degrees of post-prostatectomy incontinence, although it is not considered standard of care in irradiated patients due to lower success rates and higher complication rates [2, 46]. An adjustable MS has a higher reoperation rate, but not a higher efficacy rate, compared to non-adjustable slings [52]. A recent study analyzed decision-making in male patients with SUI in centers of expertise [53] reported that MS was less to be selected in patients with a neurological disease (5.3 vs. 9.1%; *p* = 0.030), a prior urethral stricture (22.7 vs. 50.0%; *p* = 0.001), prior incontinence surgery (24.4 vs. 45.5%; *p* = 0.01), or previous radiation therapy (26.5 vs. 40.1%; *p* = 0.001).

### What is More “Cost-Effective” - A Male Sling or An Artificial Urinary Sphincter?

There is limited published data on cost-analysis modelling comparing current continence devices with conservative non-surgical options. It is important to acknowledge that various continence devices are not commercially available or have not received governmental regulatory approval in many countries. Even in countries with access to different continence devices, these devices may not be covered by a third-party insurer and can be costly, given the out-of-pocket costs for uninsured patients.

While economic evaluation can provide a comprehensive assessment of the cost-effectiveness of surgical interventions for SUI, there is a lack of robust evidence and significant uncertainty around some parameters in such economic modelling [49]. However, it is reasonable to assume that the cost of (non-reusable) incontinence pads is an expensive ongoing expenditure, with indwelling catheters, condoms, and penile clamps having their own limitations and unique adverse issues. Moreover, there is considerable psychosocial burden and psychosexual distress related to these conservative measures. These indirect hidden costs that include visits to clinicians, lost work time, disability applications, and decreased quality of life. A study exploring the cost of SUI using claims data analysis [54] concluded that on a per capita basis, using the incremental cost-of-illness model that controls for comorbid conditions, as well as patient demographics, patients with SUI had direct costs that were 134% more than those for their controls and indirect costs that were 163% more than those for controls. More recently, another study found that [55] SUI significantly increases healthcare utilization and expenditures among prostate cancer survivors, with higher odds of above-average total expenditures (adjusted odds ratio [aOR] 2.33; 95% confidence interval [CI] 1.18–4.60; *P*=0.015), particularly through outpatient care and incontinence-related medical supplies.

Given the wide range of continence procedures available, coupled with different surgical approaches and a lack of consensus on the best continence device or surgical approach, it can be challenging to determine which continence procedure should be used to treat SUI. The MASTER trial comparing MS and AUS [56] demonstrated that MS was non-inferior to AUS (intention-to-treat estimated absolute risk difference − 0.034, 95% confidence interval − 0.117 to 0.048; non-inferiority *p* = 0.003), although MS has a lower success rate (a confidence interval excluding the non-inferiority margin of -15%). While QOL scores and satisfaction rates were high in both groups, secondary outcomes indicated better outcomes in the AUS than the MS. However, the MS costs less than the AUS, with the incremental cost-effectiveness ratio for MS compared with an AUS translating to a cost saving of £425,870 for each quality-adjusted life-year lost [54]. The MASTER trial concluded that AUS and MS are effective, although many patients continue to live with some degree of SUI postoperatively [57].

In general, it is essential to thoroughly counsel patients on the specific mechanics of different continence devices and provide informed consent regarding the unique complications associated with each procedure. The use of preoperative cystoscopy with or without a multichannel urodynamic study is often necessary to ensure that any pre-existing urological disorders are addressed and clinically stable before definitive continence surgery. Strict adherence to patient selection and safe surgical principles is critical to ensure excellent clinical outcomes and minimise surgical complications [1, 2]. Despite published guidelines advocating specific use of MS and AUS, the reality is that MS can be used in patients with severe SUI and even in the post-radiation group [58, 59], while AUS remains the more effective surgical option in men who wish to be fully continent postoperatively [2, 50, 52].

Health economic analyses have become increasingly important as a tool to help inform decisions between treatments and interventions. These cost-benefit studies provide an in-depth analysis of all relevant costs (from the specified perspective) and clinical outcomes associated with the treatments being compared, thereby helping to decide on how best to allocate resources more cost-effectively. A cost-utility study using Markov modelling to estimate the incremental cost per quality-adjusted life years (QALYs) [60] demonstrated that an AUS implantation had a 10-year mean total cost of $14 228 (SD ± 3,509) for 7.58 QALYs, while that of AdVance MS had a mean total cost $18 938 (SD ± 12,435) for 6.43 QALYs. The incremental cost savings of AUS over 10 years were -$ 4710 with an added effectiveness of 1.15 QALY, and it was likely the more economical surgical option for severe post-prostatectomy SUI.

For moderate stress SUI, a systematic review and meta-analysis comparing 5 studies (295 for slings and 214 for AUS) [61] found that AUS had a higher success rate with an odds ratio (OR) = 0.57 (95% CI: 0.36–0.90) with no significant difference observed in the overall complication rate between slings and AUS groups (OR = 1.06, 95% CI: 0.58–1.92, *P* = 0.86). Real-world data based on national database analysis [62] showed that AUS was associated with higher odds of 30-day complications (OR 1.48 (1.09–2.02), *p* = 0.012), including surgical site infections (OR 2.19), overall infections (OR 1.84), implant complications (OR 4.08), genitourinary complications (OR 2.31), unplanned reoperation (OR 2.04), Clavien-Dindo Grade I-II (OR 1.58) and Grade III complications (OR 2.10), and prolonged hospital stay (OR 4.66–5.71; all *p* < 0.001) compared to MS placement. Using the American College of Surgeons National Surgical Quality of Improvement Program, another study [63] found MS placement had a lower 30-day postoperative complication rate compared to AUS (2.8 vs. 5.1%, *p* = 0.046) and that patients with higher BMI were more likely to be associated with complications. A large multicentre European study analysing failure rates of MS and AUS [64] reported that prior history of pelvic irradiation was an independent risk factor for explantation in AUS (*p* < 0.001) and MS (*p* = 0.018) as well as statistically significantly higher rates of urethral stricture and erosion. Another retrospective multi-institutional study [59] found that AUS patients, in comparison to MS patients, had a significantly lower mean pad usage during daytime (*p* < 0.001) and nighttime (*p* = 0.018) as well as a higher subjective complete dry rate (57.3% vs. 22.0%; *p* < 0.001). This is in spite AUS patients had more complex prior history and pathogenesis of urinary incontinence as well as a more severe grade of SUI.

Another recent meta-analysis and systematic review on AUS surgery [65] of a pooled data of 1271 patients from 19 studies (6 prospective cohort studies, 12 retrospective cohort studies, and 1 randomized controlled trial) concluded that AUS significantly decreased the number of pads used (pads/ day) by about 4 (*p* < 0.001) with higher quality of life outcomes (*p* < 0.001) although the authors acknowledged considerable heterogeneity in definition of continence and limited follow-up.

Prevention of SUI is ideal if the underlying sphincteric complex and pelvic floor muscles can be safeguarded during the course of prostate cancer treatment. However, the reality is that damage to the continent mechanisms can occur with any form of prostate cancer treatment, despite the best technology and pre-treatment optimization. Shared decision-making, patient-specific considerations, and appropriate postoperative monitoring remain critical to optimizing outcomes and patient satisfaction rates. Novel and new continence devices should be innovative in concept and function, simulating a normal human sphincter that can respond pre-emptively and effectively during physical activity. Nanotechnology-driven devices and cellular-based therapy are exciting and likely transformative, but they are not currently commercially available and have limited published data in terms of clinical efficacy, durability or safety. Until the emergence of a better-engineered continence device, and/or more robust, proven cellular-based therapy or tissue engineering, the quest for an ideal urinary continence therapy remains elusive.

## Conclusions

Presently, significant gaps exist in the cost analysis and modelling for the most effective treatment in male SUI. While AUS has been considered by many as the standard of care in male SUI, MS is rapidly emerging as a valid and simpler alternative. The actual cost for continence surgery can vary depending on healthcare provision and private insurance, but from a cost analysis, continence surgery is more cost-effective than wearing incontinence pads. Complications following continence surgery are uncommon, and the overall quality of life outcomes are better in patients who are continent. Regardless of the surgical intervention, shared decision-making should be undertaken in patient counselling with emphasis on personalised care and what is best for each individual patient living with SUI.

## Data Availability

No datasets were generated or analysed during the current study.
